# Age and gender differences in Brazilian adolescents’ vocational interests

**DOI:** 10.3389/fpsyg.2025.1498424

**Published:** 2025-03-19

**Authors:** Filip De Fruyt, Ana Carla Crispim, Gustavo Henrique Martins, Ricardo Primi, Rodolfo Augusto Matteo Ambiel, Joyce Scheirlinckx, Oliver P. John

**Affiliations:** ^1^Departament of Developmental, Personality and Social Psychology, Ghent University, Ghent, Belgium; ^2^eduLab21, Institute Ayrton Senna, São Paulo, Brazil; ^3^Institute Ayrton Senna Chair, Ghent University, Ghent, Belgium; ^4^Department of Psychology, Universidade São Francisco, Campinas, Brazil; ^5^Post Graduate Program in Psychology, Universidade São Francisco, Campinas, Brazil; ^6^Post Graduate Program in Psychology, Pontifícia Universidade Católica de Campinas, Campinas, Brazil; ^7^Departament of Psychology and Institute of Personality and Social Research, University of California, Berkeley, Berkeley, CA, United States

**Keywords:** vocational interests, RIASEC, interests development, interests assessment, educational assessment

## Abstract

This study examines age and gender differences in a large cross-sectional sample of Brazilian middle and high school students (Total N = 234.857) who were administered 18REST-2, a brief RIASEC measure used in large-scale educational monitoring. Paralleling to previous meta-analytic findings, boys scored substantively higher on Realistic, whereas girls scored higher on Social and Artistic interests. Enterprising interests were greater among older adolescents, plateauing after middle school, also in line with meta-analytic work, but also conventional interests were greater across adolescence, counter to meta-analytic patterns. Similarities and differences with meta-analytic findings are discussed considering longer versus brief measures to assess RIASEC, the different cultures in which studies are conducted (Brazil versus mainly Western countries) and the more recent nature of the present data reflecting the post-pandemic era.

## Introduction

The past decade, the construct of vocational interests regained attention in both education and human resources (Wille and De Fruyt, 2023). In educational counseling psychology, vocational interests are presumed to be critical factors determining in what kind of academic majors students will enroll (Schelfhout et al., 2021). In addition to their relevance for educational-choice behavior, vocational interests, more recently, also showed to be predictors of both continuance in studying and academic achievement (Nye et al., 2012).

For more than half a century, John Holland’s model of vocational interests (Holland, 1965, 1997) guided both research and professional counseling practice. The model suggests that vocational interests of individuals can be described using six vocational interest domains, i.e., Realistic (R), Investigative (I), Artistic (A), Social (S), Enterprising (E) and Conventional (C). These same interest domains can be also used to describe environments that individuals prefer, such as study majors or occupational environments. The RIASEC model was further used as an organizing framework in various meta-analyses to document gender (Su et al., 2009) and age differences (Hoff et al., 2018), describe developmental trajectories of RIASEC dimensions (Low et al., 2005), and investigate their predictive validity (Nye et al., 2012). More recently, alternative models to represent vocational interests have been proposed, such as the SETPOINT model by Su et al. (2019) to better accommodate new jobs.

Supporting educational choice processes and monitoring academic performance became a top priority for policy makers, to ensure that students are well prepared for the challenges of the quickly changing labor market. Age and gender turned out as critical demographic characteristics to understand students’ vocational interests, given that vocational interests already develop early-on (Renninger et al., 2019) and the underrepresentation of girls in STEM (Science, Technology, Engineering, & Math) majors (Su et al., 2009; Su and Rounds, 2015).

### Gender and age differences in interests

It is a well-established finding that boys and girls differ in their mean scores on vocational interest domains. Su et al. (2009) meta-analytically described the gender differences that are typically found in RIASEC research summarizing findings obtained from 81 samples including 243,670 men and 259,518 women, with the mean ages of the samples varying from 12.50 to 42.55 years. Men have, on average, substantively higher scores on Realistic (*d*-effect size = 0.84) and somewhat higher scores on Investigative (*d* = 0.26), whereas women are on average higher on Social (*d* = 0.68), Artistic (*d* = 0.35) and Conventional (*d* = 0.33) interests. They did not find differences for Enterprising. Moderator analyses suggested that gender differences were largest in younger groups. The authors concluded that vocational interests may play an important role in explaining gendered educational and occupational choices such as the gender disparity observed among students enrolled in STEM fields.

Rank-order stability of vocational interests has been examined in detail by Low and colleagues (Low et al., 2005). This type of stability describes the relative ordering of individuals in terms of interests across time, typically expressed by a correlation coefficient. This form of stability has to be distinguished from mean-level or normative changes that describe average scores of a group of individuals across time. Both types of stability can be independent, i.e., the average for a group or cohort can change across time, but relative ordering of individuals may be kept preserved longitudinally (De Fruyt et al., 2006).

Low et al. (2005) quantitatively examined 66 longitudinal studies from adolescence to adulthood. The rank-order stability of interests was already substantive during adolescence (ages 12–18) with adjusted stability coefficients in-between 0.55 and 0.58. Coefficients increased profoundly during the college years (ages 18–24) mounting to 0.70, and continued to increase during young adulthood, to values around 0.80 for the age group 25 to 30 years. Overall, these findings suggest that vocational interests are more stable than personality traits. These correlation findings, however, are not informative on the direction of change (increasing or decreasing, linear or nonlinear, etc), their magnitude, and potential gender differences in such patterns (Hoff et al., 2018).

Mean-level or normative change of vocational interests has been meta-analytically described by Hoff et al. (2018) examining 49 longitudinal studies containing 98 total samples and 20,639 participants, spanning early adolescence (~11 years; middle school years) to mid-adulthood (~age 42). Early adolescents (11–14 years; *N* = 986) were least represented, whereas most data were available for adolescents (14–18 years; *N* = 10,285). Overall, mean interest scores across RIASEC slightly increased over time, though with differences for specific RIASEC domains: levels of artistic, social and enterprising interests (the ‘People’ dimension) slightly increased over time, whereas interests directed at ‘Things’ stayed equal (i.e., realistic and investigative) or means slightly decreased (i.e., conventional). When examining change patterns across age categories, they found that interest intensity decreased by about 1/10th of a standard deviation in middle school (*d* = −0.11), subsequently increasing during high school (*d* = 0.08). Average interest intensity remained largely unchanged during the college years and emerging adulthood with *d*’s = 0.01 and 0.02, respectively, (both not significant), although there were differences for specific RIASEC dimensions.

The drop in mean-level change scores observed in middle school (early adolescence), followed by an increase in high school is largest for Conventional (decline in middle school of *d* = −0.30), followed by an increase (*d* = 0.06) in late adolescence, and similar patterns can be observed for Realistic and Social interests. There is an exception for Enterprising, however, showing a steadily increasing pattern across early and late adolescence. There were notable gender differences in mean-level changes during middle school for both realistic and social interests. Realistic interests of both boys and girls decreased, but those for girls decreased more steeply. Social interests of girls increased slightly, whereas those of boys declined steeply. Hoff et al. (2018) concluded that gender differences for vocational interests widen during early adolescence.

### Present study

The primary studies used for the gender (Su et al., 2009) and age (Hoff et al., 2018) meta-analyses described above administered a variety of measures assessing RIASEC domains usually with an extended set of items. Moreover, most studies in the meta-analyses were conducted at least 10 years ago, and assessed mainly populations from Majority World countries. The present study adds to these findings and will investigate vocational interests in a large sample (*N* = 234.857) of Brazilian middle and high school students who were administered a brief RIASEC measure as part of a large-scale survey conducted in 2021, shortly after the pandemic. Our work extends thus the meta-analytic research investigating (a) whether observed gender differences and age patterns are also replicable with a brief RIASEC measure used in large-scale monitoring surveys, and (b) whether these patterns also replicate in a non-Western student sample. We further examine (c) whether the reported gender and age patterns are still valid today, in a new socio-economic reality after the pandemic, characterized by an increase of working remotely, a range of new job titles and a changed set of required skills.

## Method

### Participants

In 2021, data on vocational interests were collected as part of a broader monitoring study conducted in schools in the Southeast of Brazil on behalf of the State Secretariat of Education. Students were invited to participate as part of the educational assessment program at school with over a million students enrolled (*N* = 1.065.238 students). The study was developed as a Balanced Incomplete Block (BIB) design, thus, each student answered only part of the research protocol. In total, we excluded 370.833 students who did not answer any questions of the survey and/or valid booklets.

Vocational interests were assessed in four out of 12 booklets of the research protocol with BIB design (*N* = 252.795 students). Additionally, *N* = 17.938 were removed due to failing the carelessness detection filter, which means that they provided the same response consecutively in 15 or more items. Thus, data for a group of *N* = 234.857 students enrolled in *N* = 7,460 public schools were finally available for analyses. Gender distribution was *N* = 119.248 girls (50.77%) and *N* = 115.609 boys (49.23%). Students were either in the 5th grade (*N* = 68.568), 9th grade (*N* = 90.298) or in the 12th grade (*N* = 75.991). Information on age was not available, but given that school grades are distant, overlap due to age-grade distortion is less likely to be the case. The expected ages for each grade are 5th grade = 11 years old; 9th grade = 14 years old; and 12th grade = 17 years old. Information on students’ social-economic status was only available at the school level using a governmental index called INSE (i.e., Socio Economic Level Index). Self-reports by students on social-economic indicators turned out to be unreliable for this group, given that many students do not know the parental income or the education level of their parents. Categories in INSE closer to 1 are related to schools with more families that have less material and educational resources, while categories closer to 7 are related to schools with more families that have more material and educational resources. Various social-economic backgrounds were represented in our sample, with probably an overrepresentation of middle-economic status groups, such as category 4 (10%) and category 5 (68.7%), which combined represent around 79% of the sample investigated.

### Measure and procedure

RIASEC vocational interests were assessed with 18REST–2 (Martins et al., 2024), an inventory specifically designed to be used in large-scale surveys when only limited assessment space is available. 18REST-2 uses 3 items to assess each RIASEC domain. Items are presumed to reflect the breadth of their respective construct domains, so lower internal consistencies are to be expected. Cronbach alpha reliabilities in the current sample were 0.70 (R), 0.64 (I), 0.69 (A), 0.58 (S), 0.73 (E), and 0.66 (C), considered satisfactory for scales with a small number of items (see also Soto and John, 2019 and Baardstu et al., 2025 for internal consistencies of short personality scales).For short measures, construct breadth coverage and test–retest reliability are more important than internal consistency (McCrae and Mõttus, 2019).

Assessments were conducted paper and pencil during classroom hours and were done on a voluntary basis and anonymously. Approval for design and data collection was provided by an Ethical Review Board.

RIASEC items were administered together with items assessing other psychological constructs. To keep the number of items manageable, a Balanced Incomplete Blocks (BIB) design was used, with twelve booklets (labeled from A–K). Each participant was required to answer only one booklet. 18REST-2 items were represented in four booklets, i.e., in A, C, H and J. The distribution of items in each booklet and the number of participants for each can be obtained from the authors. 18REST-2 item scores were aggregated into respective RIASEC sum scores.

All data analyses were performed in R Studio. Mean differences were tested using t-tests. The magnitude of the differences were explored using Cohen’s d.

## Results

We first examined mean RIASEC scores for boys and girls in the total sample (see Table 1). There were significant gender differences for five of the six vocational interest domains. Boys were considerably higher (*d* = 0.58, *p* < 0.0001) on Realistic, whereas girls were substantively higher on Artistic (*d* = 0.43, *p* < 0.0001) and Social (*d* = 0.36, *p* < 0.0001) with minor to negligible differences for Investigative (*d* = 0 0.13, *p* < 0.0001) and Enterprising (*d* = 0.05, *p* < 0.0001) respectively. There were no significant differences for Conventional.

**Table 1:** RIASEC mean scores and standard deviations by gender for the total sample.

	Girls		Boys				
	*M*	*SD*	*M*	*SD*	*t*	*p*	*d*
Realistic	2.00	0.84	2.53	0.99	−133.066	<0.01	0.58
Investigative	2.96	0.99	2.82	1.00	30.656	<0.01	0.13
Artistic	2.60	1.05	2.17	0.96	98.053	<0.01	0.43
Social	3.50	0.85	3.18	0.86	82.897	<0.01	0.36
Enterprising	2.82	1.00	2.88	1.02	−12.081	<0.01	0.05
Conventional	2.66	0.98	2.66	0.97	−0.651	0.52	0.00

An overview of students’ mean RIASEC scores, split for boys and girls, in 5th, 9th and 12th grade are described in Table 2. For realistic interests, the gap between boys and girls grows from 5th grade (*d* = 0.21), over 9th grade (*d* = 0.60) to 12th grade (*d* = 0.88). The averages for boys get larger across grades, whereas the means for girls become smaller.

**Table 2:** RIASEC mean scores and standard deviations by gender and school grade.

		Girls		Boys				
Interest	Grades	*M*	*SD*	*M*	*SD*	*t*	*p*	*d*
Realistic	5th	2.15	0.88	2.35	0.99	−25.27	<0.01	0.21
	9th	2.01*	0.82	2.54*	0.94	−85.41	<0.01	0.60
	12th	1.87*	0.82	2.67*	1.01	−116.77	<0.01	0.88
Investigative	5th	3.04	1.01	2.88	1.05	18.79	<0.01	0.16
	9th	2.98*	0.98	2.78*	0.98	29.42	<0.01	0.20
	12th	2.86*	0.98	2.83*	0.99	4.39	<0.01	0.03
Artistic	5th	2.77	1.05	2.32	0.99	52.05	<0.01	0.44
	9th	2.53*	1.02	2.05*	0.90	72.23	<0.01	0.50
	12th	2.54	1.08	2.18*	1.00	46.45	<0.01	0.35
Social	5th	3.40	0.87	3.21	0.89	24.48	<0.01	0.21
	9th	3.42*	0.84	3.11*	0.83	53.51	<0.01	0.37
	12th	3.66*	0.84	3.24*	0.86	64.31	<0.01	0.48
Enterprising	5th	2.55	0.99	2.52	1.01	3.79	<0.01	0.03
	9th	2.87*	0.97	2.92*	0.98	−8.47	<0.01	0.06
	12th	2.98*	1.01	3.10*	1.01	−15.59	<0.01	0.12
Conventional	5th	2.44	0.97	2.42	1.01	2.86	<0.01	0.02
	9th	2.68*	0.94	2.67*	0.93	0.93	0.35	0.01
	12th	2.80*	0.99	2.83*	0.94	−4.93	<0.01	0.04

Asterisks represent significance level testing (*p* < 0.01) between the current school grade in comparison to the previous school grade. For school grades significance level testing, the significance levels represent differences between 5_th_ and 9_th_ school grades, and 9_th_ and 12_th_ school grades.

For investigative, means for girls are slightly higher in 5th and 9th grade, but equal in 12th grade. Mean scores are slightly lower with increasing grades for girls.

Across school grades, a consistent gender difference is observed, with girls scoring higher than boys, with respective d’s of 0.44 (5th grade), 0.50 (9th grade) and 0.35 (12th grade). The means for girls in 9th and 12th grade are slightly lower than in 5th grade. The mean for boys shows a small dip in 9th grade.

Social interests are already slightly higher in 5th grade (*d* = 0.21) for the girls, and this gender difference further widens across 9th (*d* = 0.37) and 12th (*d* = 0.48) grade. Average social interest scores for girls are slightly larger across grades, whereas a small dip is shown for boys in 9th grade.

For each grade, gender differences for Enterprising are negligible with d’s of 0.03, 0.06 and 0.12, respectively. For enterprising, larger averages are observed across grades for both boys and girls.

Finally, and paralleling findings for enterprising interests, conventional interests show negligible gender differences across grades, with larger averages across grades for boys and girls.

## Discussion

The present study complements the impressive meta-analytic work by Su et al. (2009) and Hoff et al. (2018) investigating gender and age differences in a large convenience sample of students attending various types of education in Brazil in the post-pandemic era. Students were assessed at the critical time of adolescence in 5th, 9th and 12th grades in which students make first educational choices impacting their future steps on the labor market. Assessments were conducted after the pandemic using a brief RIASEC measure that is used for educational monitoring purposes. Data collected in South America are usually underrepresented in academic research, and the present study fills this gap, enabling to compare developmental patterns from a large Brazilian sample to patterns mainly derived from North American and European samples assessed before the pandemic. Studies like this are necessary to examine whether patterns generalize to countries with conditions like Brazil, characterized by a high rate of social inequality, which affects directly the opportunities and activities that students have access to during their lives. It is hence critical to examine developmental vocational interest patterns in these cultures, to strengthen or nuance findings learned from the meta-analytic work. Moreover, our study has large and sufficient numbers of participants in pre-, middle and late adolescence, to provide a stable account of mean-level changes of vocational interests across full adolescence, counter to Hoff et al. (2018) who had less than 1,000 pre-adolescents.

Overall, our results by gender and grade showed similar patterns like reported in the meta-analyses by Su et al. (2009) and Hoff et al. (2018). In line with Su et al. (2009), Brazilian boys scored substantively higher on realistic, whereas girls scored higher on social and artistic, though magnitudes of differences were smaller. In addition, no gender differences for enterprising were observed. In contrast to Su et al. (2009), Brazilian boys and girls did not differ on conventional, and boys (instead of girls) scored slightly lower on Investigative.

The present analyses further confirm the more specific mean-level normative patterns in pre- and late adolescence described by Hoff et al. (2018) during pre-pandemic times, except for conventional that showed an increase from 5th to 9th grade instead of a decline. In line with Hoff et al. (2018), a similar increase from 5th to 9th grade for both boys and girls was observed for enterprising, slightly continuing to 12th grade. Also in our work, there were notable gender differences in mean-level changes during middle school for both realistic and social interests. In line with Hoff et al. (2018) realistic interests of girls decreased, but in our sample boys’ realistic interests increased showing the largest gender gap in 12th grade. In both Hoff et al. (2018) and our study, Social interests of girls increased slightly to 12th grade, but showed a small dip for boys in 9th grade. Counter to Hoff et al. (2018) gender differences for vocational interests in our sample seem to widen during late instead of early adolescence, except for enterprising and conventional interests that showed no gender differences across grades.

Various theories have been suggested to explain gender differences in vocational interests and how these develop, ranging from biological to social psychological theories. Studies by Berenbaum (1999), for example, suggest that differences in androgens already affect interests early-on in development, whereas Eccles (1994) used an expectancy-value model to explain differential gender preferences for educational and occupational choices. Additionally, various studies underscored the influence of gender roles (Eagly, 1987) and socialization processes (Jacobs et al., 2005) on interest levels of boys and girls. Expectations of parents, and vocational interests and professional activities of parents themselves affect what boys and girls prefer, but may also dislike in some cases.

Unique features of this work are the large sample of Brazilian students equaling almost half of the total number of participants enclosed in the meta-analysis of Su et al. (2009). Limitations are the cross-sectional nature of the data preventing a continuous examination of the development of vocational interests across 5th, 9th and 12th grade. The availability of longitudinal data, preferably with multiple assessment points, would have allowed the study of stability and change in vocational interests across adolescence. In addition to several resemblances with the results obtained by Hoff et al. (2018), we also observed notable differences. Such deviations could be due to several factors, including the specific brief measure used in the present study, sample differences associated with the region and country in which data were collected, or time and cohort differences, reflecting a changed socio-economic reality after the pandemic. Cultures may differ in the degree to which gender roles are emphasized, or the magnitude of gender differences may be affected by dimensions of culture, such as masculinity-femininity (Hofstede, 1998). Although Brazil has the 9th largest gross domestic product (GDP) in the world and has the 8th largest purchasing power parity in the world (International Monetary Fund, 2023), the country faced specific challenges that may have affected youth differently and specifically, such as large drop out numbers in education, high violence rates and temporary closure of schools for a long time during the pandemic.

Overall, the present work has made clear that it is always necessary to specifically examine age and gender differences and compare to meta-analytic findings. The patterns described here complement the meta-analytic findings better detailing vocational interest mean-level changes during the critical period of adolescence.

## Data Availability

The data analyzed in this study is subject to the following licenses/restrictions: the dataset presented in this article is not readily available because of legal and privacy reasons in the Brazilian laws. Requests to access these datasets should be directed to Ana Crispim, acrispim@ias.org.br.
